# Supplementary material for: In Situ Aqueous Spice Extract-Based Antifungal Lock Strategy for Salvage of Foley’s Catheter Biofouled with Candida albicans Biofilm Gel
Source: Gels. 2025 Jan 1;11(1):23. doi: 10.3390/gels11010023 (PMC11765466; doi:10.3390/gels11010023)
Supplement: Supplementary file 1 [file gels-11-00023-s001.zip › Supplementary material 1.pdf]

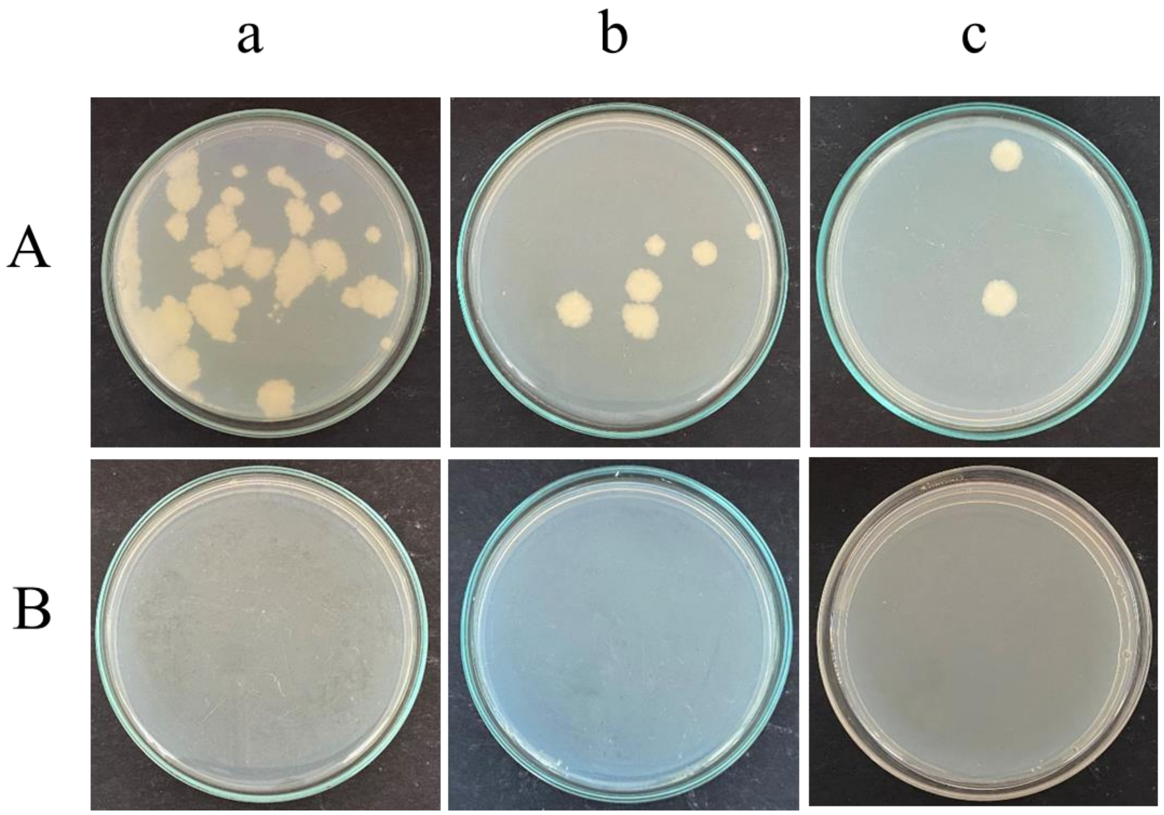

Figure S1: CFU of (A) *C. albicans* M-207 control treated with (B) garlic extract at 12 h of incubation. (a) 10^{-2}, (b) 10^{-3}, (c) 10^{-4} dilutions

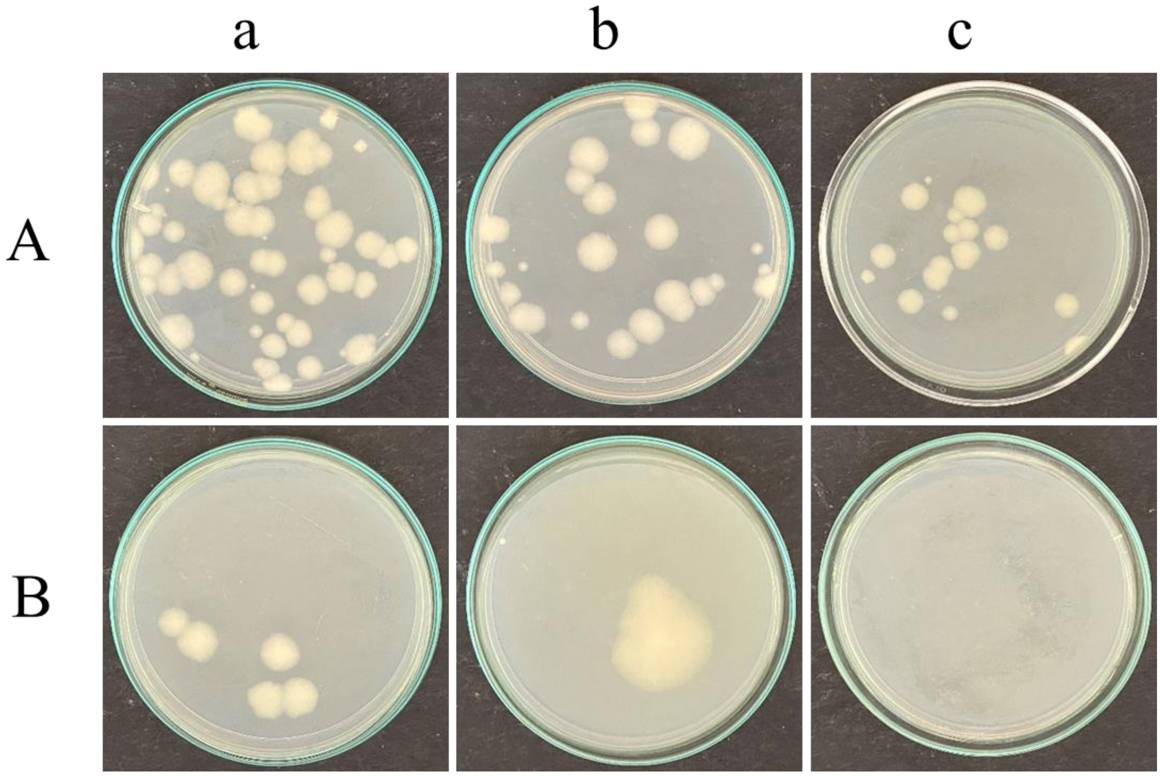

Figure S2: CFU of (A) *C. albicans* M-207 control treated with (B) garlic extract at 24 h of incubation. (a) 10^{-2}, (b) 10^{-3}, (c) 10^{-4} dilutions

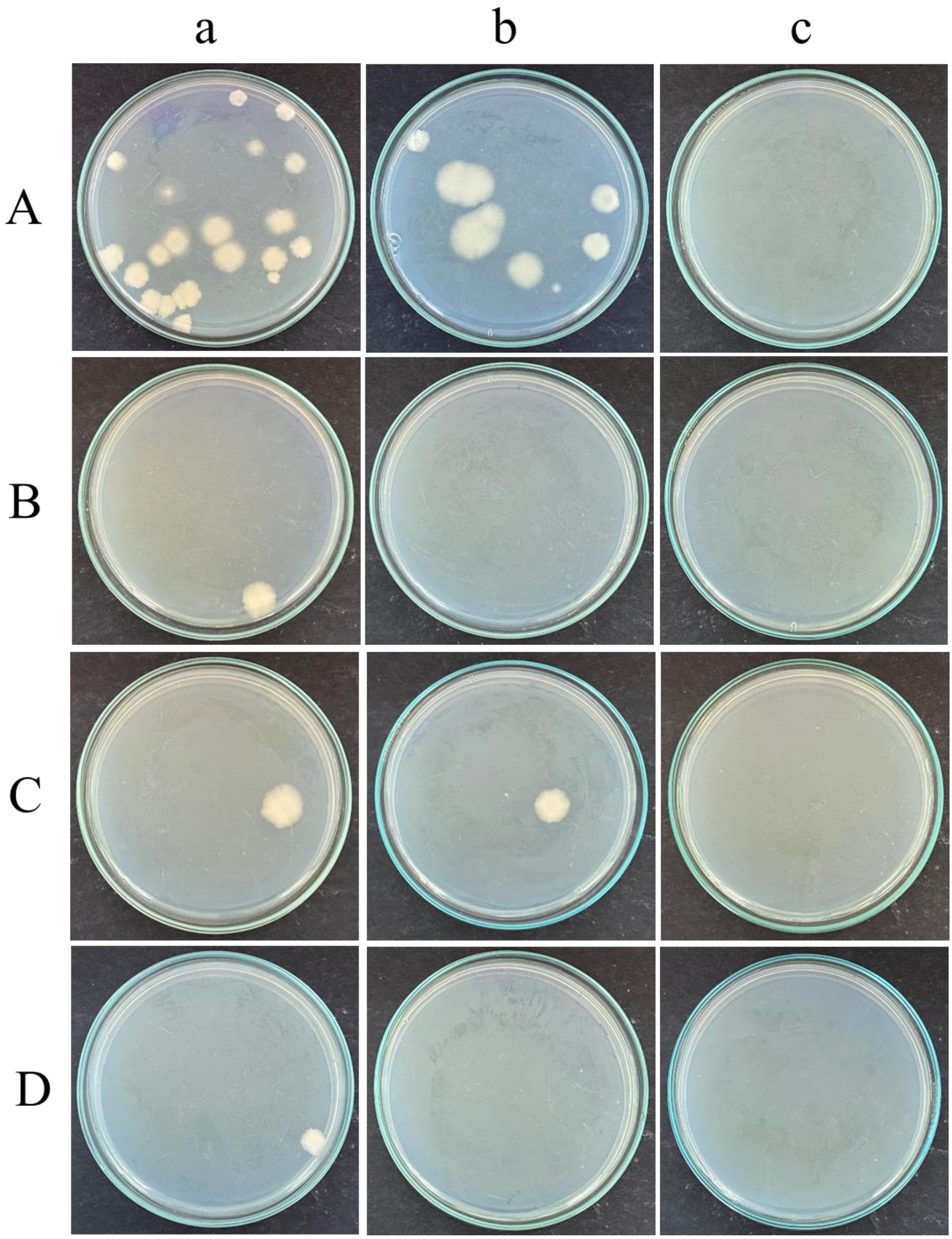

Figure S3: CFU of (A) *C. albicans* S-470 control treated with (B) garlic, (C) gooseberry, and (D) clove extracts at 12 h of incubation. (a) 10^{-2}, (b) 10^{-3}, (c) 10^{-4} dilutions

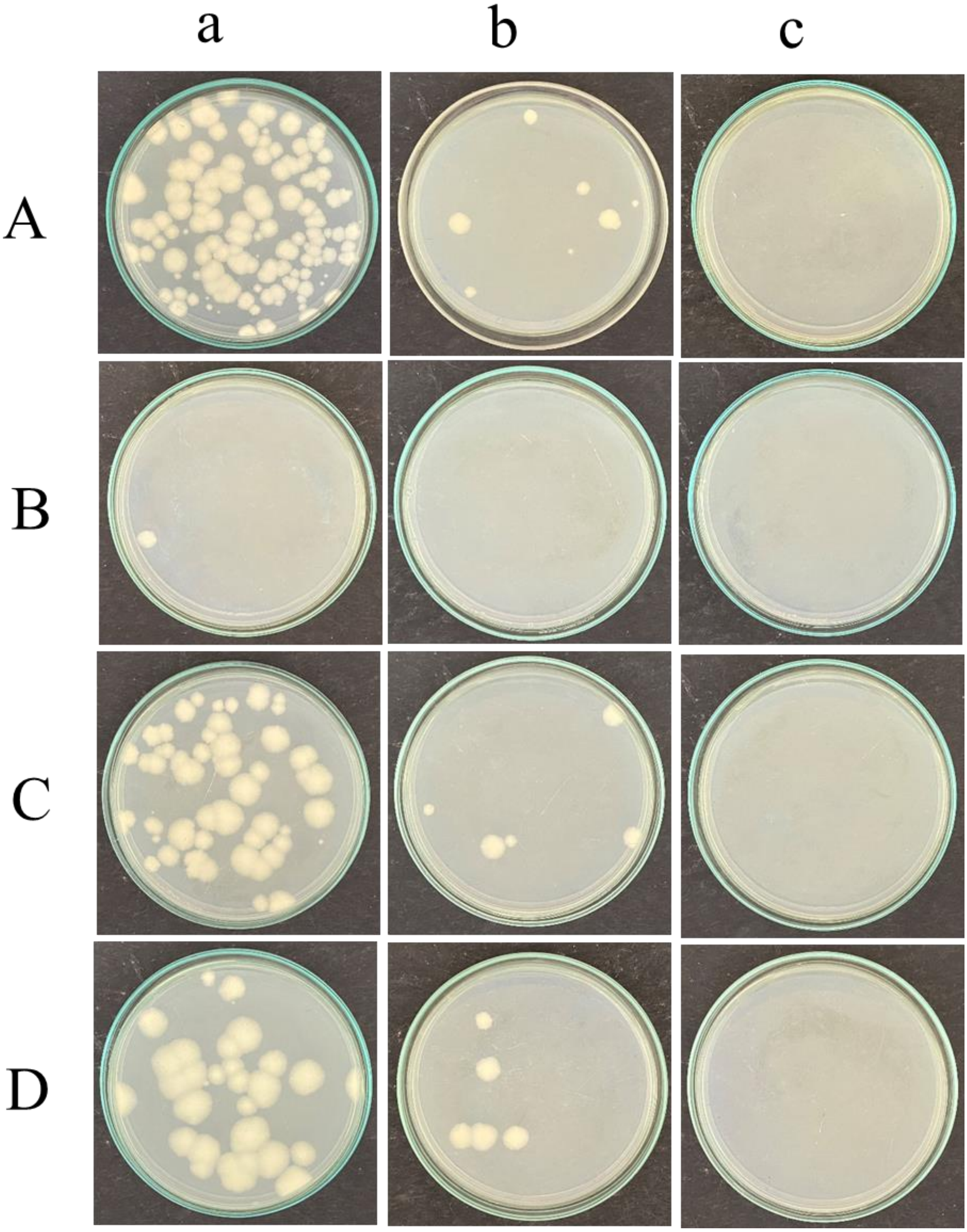

Figure S4: CFU of (A) *C. albicans* S-470 control treated with (B) garlic, (C) gooseberry, and (D) clove extracts at 24 h of incubation. (a) 10^{-2}, (b) 10^{-3}, (c) 10^{-4} dilutions

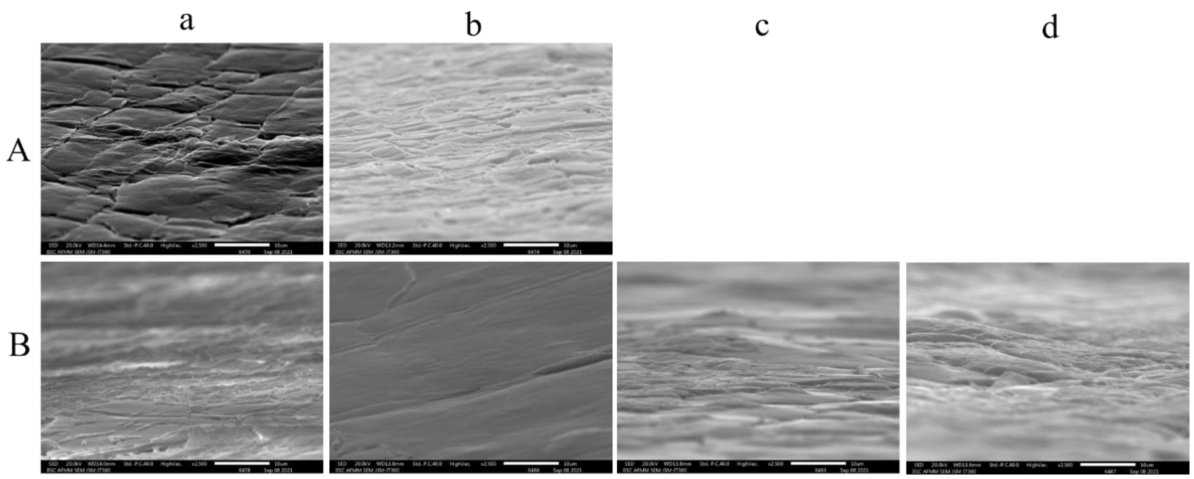

Figure S5: SEM analysis of cross section of catheter for (A) *C. albicans* M-207 and (B) *C. albicans* S-470 at 12 h. (a) Control, (b) Garlic treated, (c) Gooseberry Treated, (d) Clove treated.

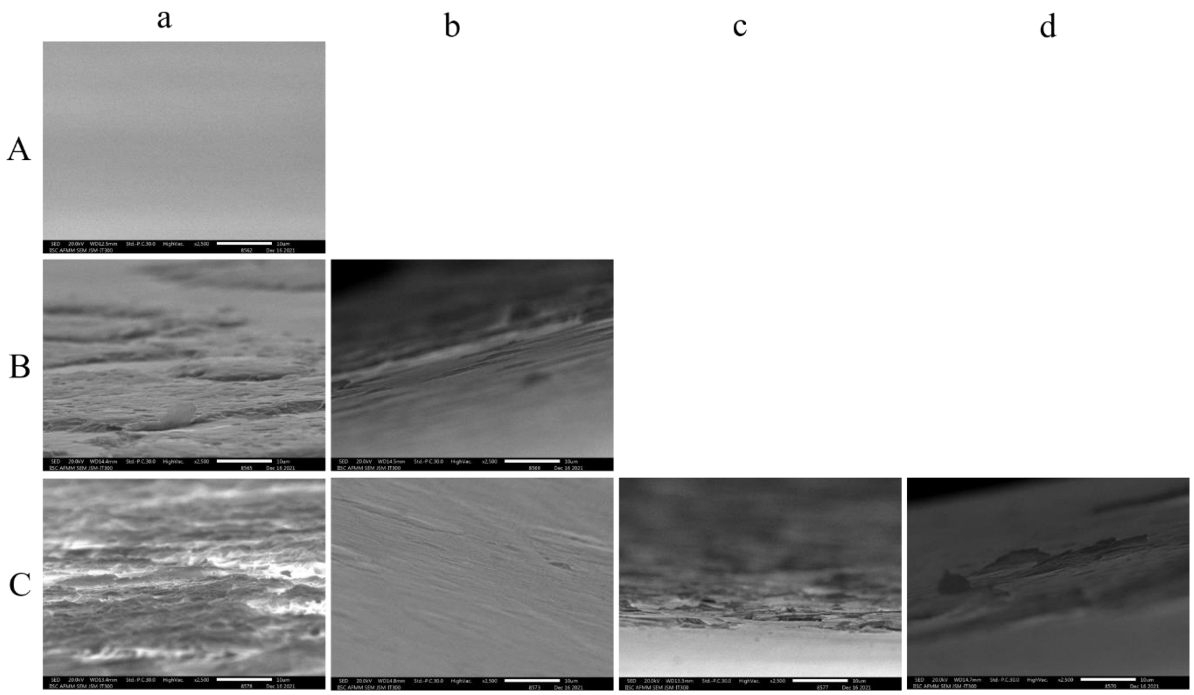

Figure S6: SEM analysis of cross section of catheter for (A) Blank, (B) *C. albicans* M-207 and (C) *C. albicans* S-470 at 24 h. (a) Control, (b) Garlic treated, (c) Gooseberry Treated, (d) Clove treated.
