# Supplementary material for: In Situ Aqueous Spice Extract-Based Antifungal Lock Strategy for Salvage of Foley’s Catheter Biofouled with Candida albicans Biofilm Gel
Source: Gels. 2025 Jan 1;11(1):23. doi: 10.3390/gels11010023 (PMC11765466; doi:10.3390/gels11010023)

Enlarged images of Figure 4, Figure S5 and Figure S6

Figure 4

PANEL 1: Blank Catheter

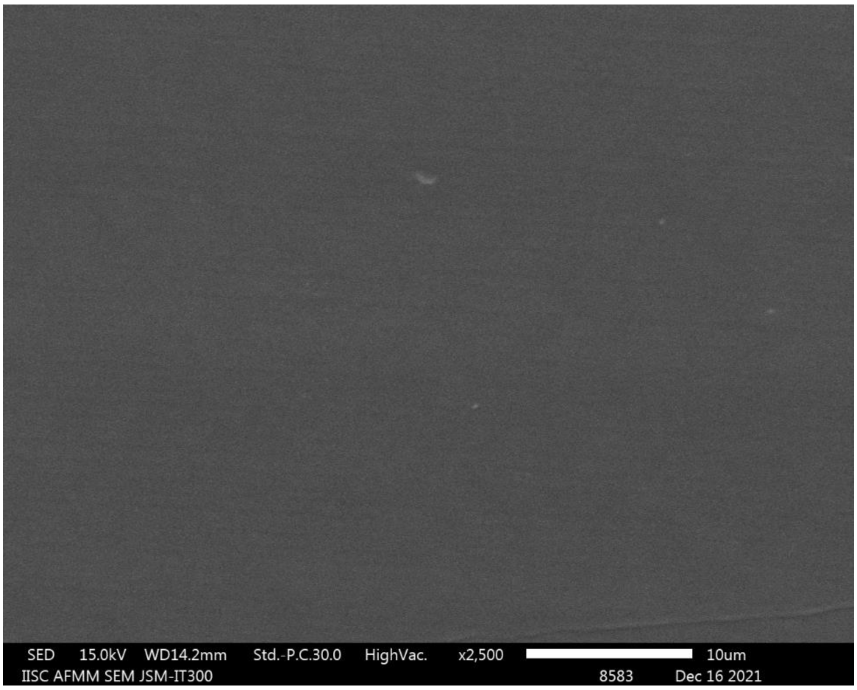

PANEL 2

(Aa) *C. albicans* M207 Control at 12 h

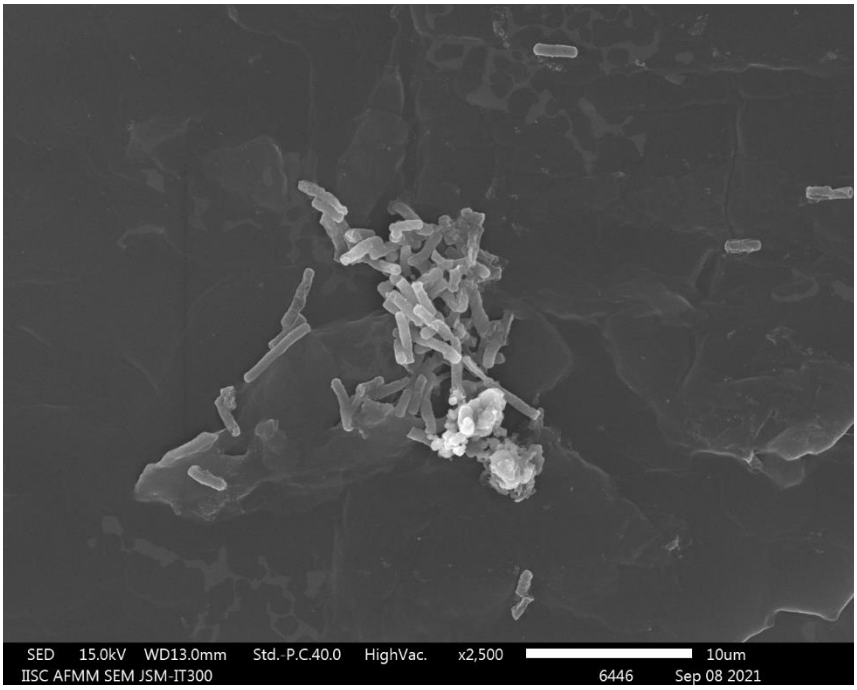

(Ab) *C. albicans* M207 Garlic Treated at 12 h

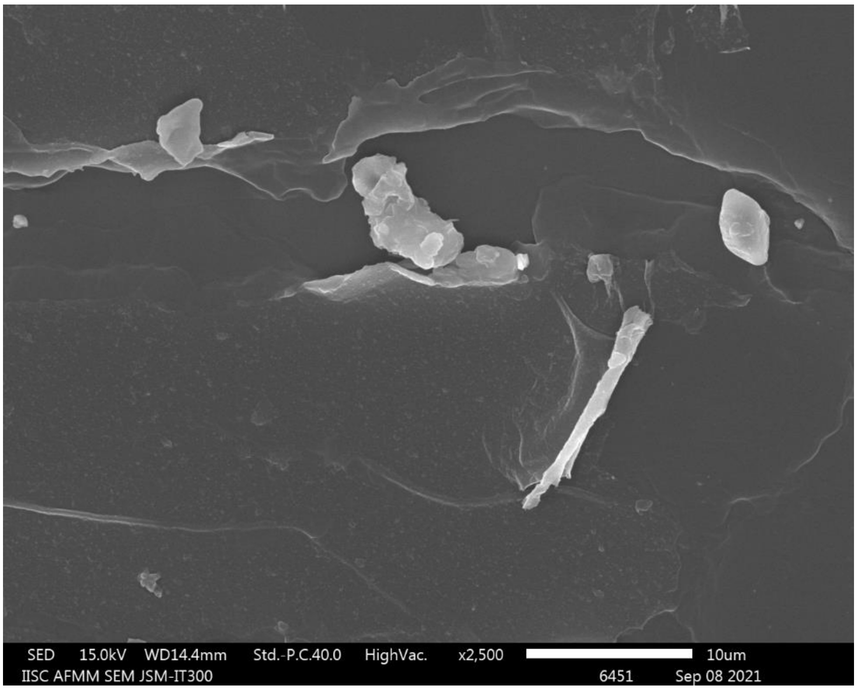

(Ba) *C. albicans* S470 Control at 12 h

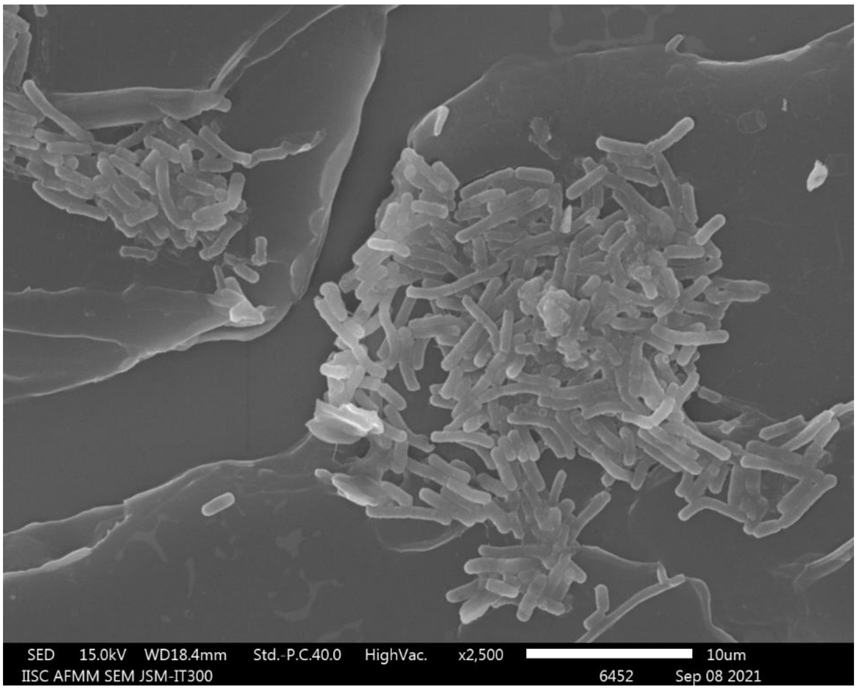

(Bb) *C. albicans* S470 Garlic Treated at 12 h

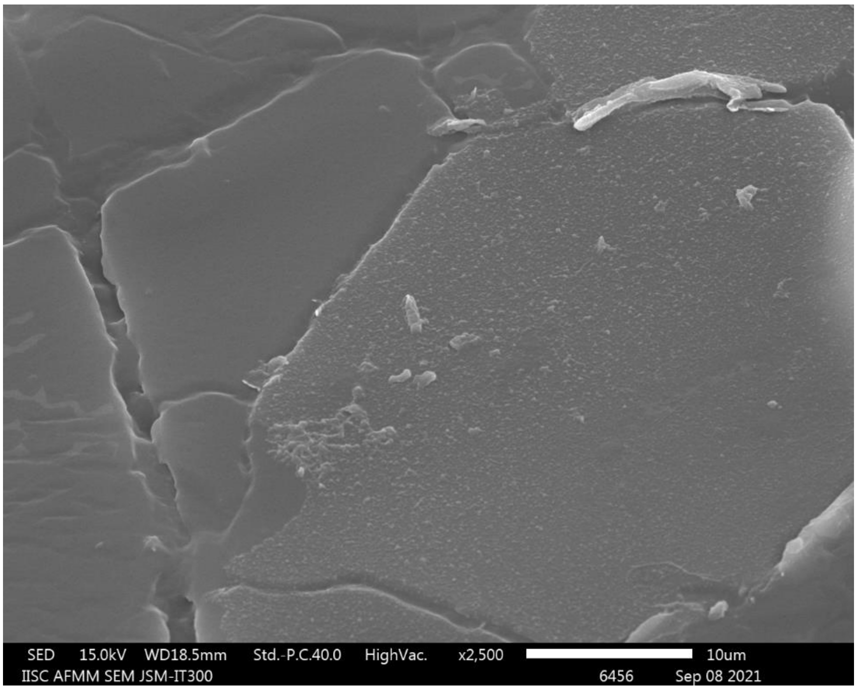

(Bc) *C. albicans* S470 Gooseberry Treated at 12 h

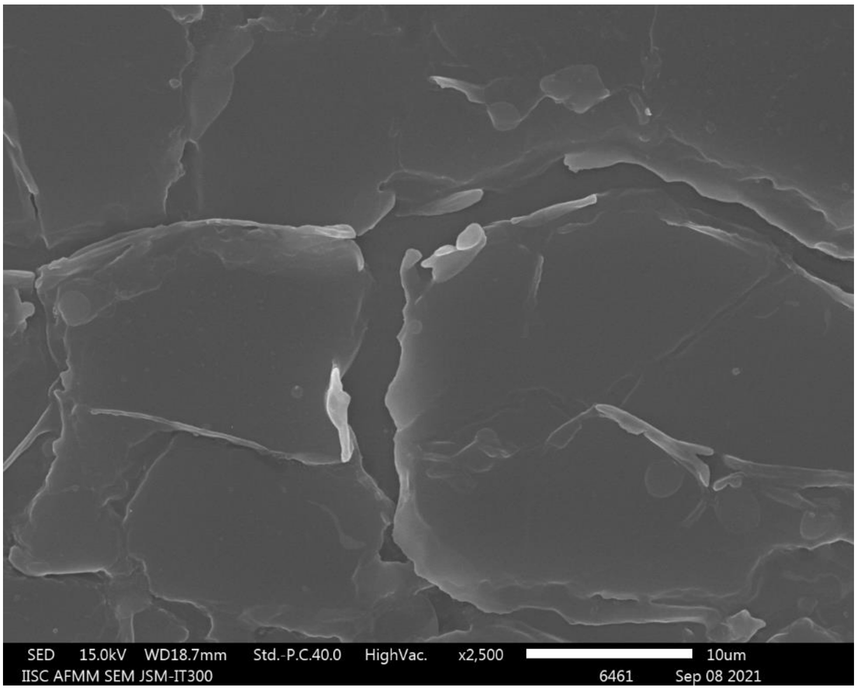

(Bd) *C. albicans* S470 Clove Treated at 12 h

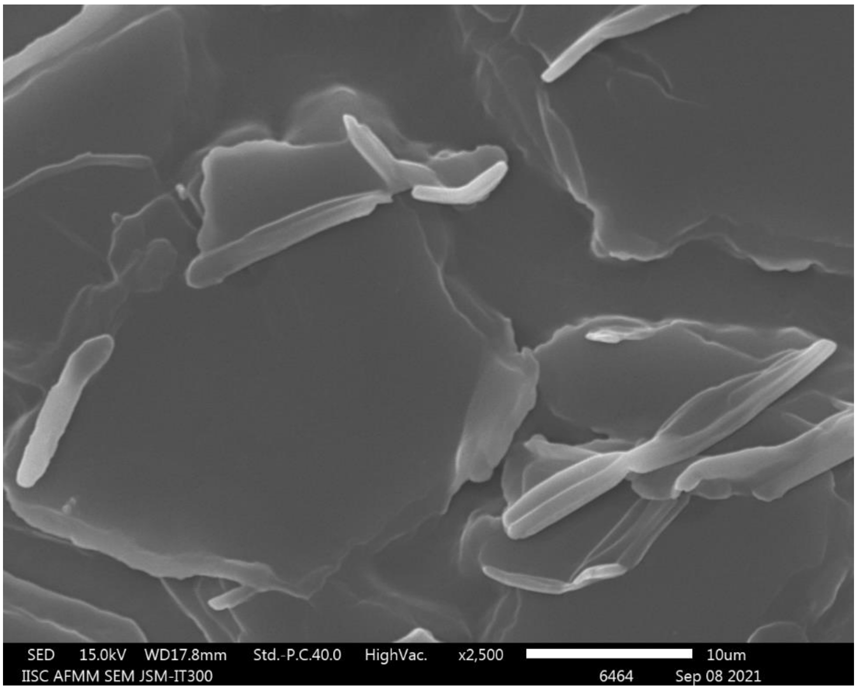

(Ca) *C. albicans* M207 Control at 24 h

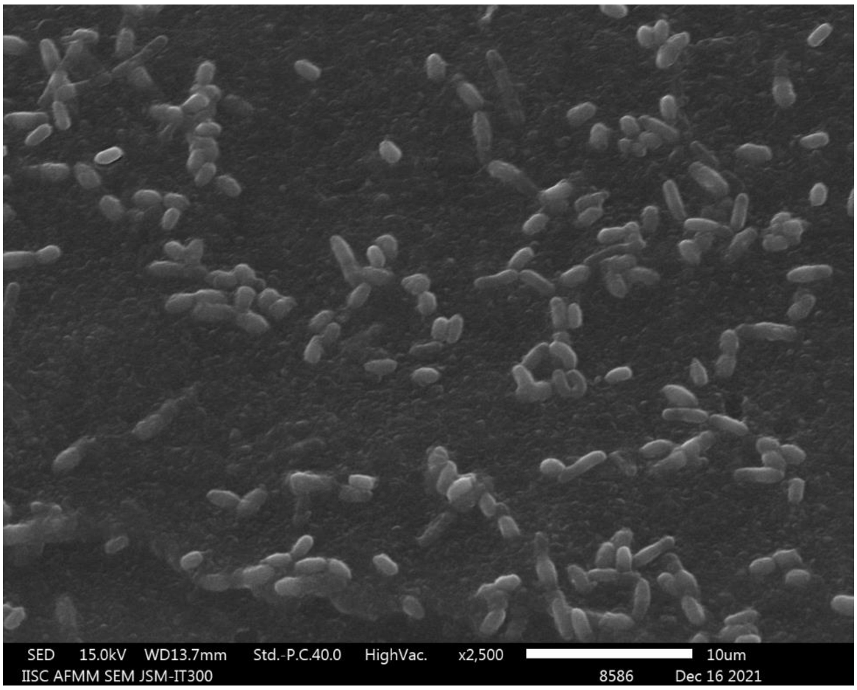

(Cb) *C. albicans* M207 Garlic Treated at 24 h

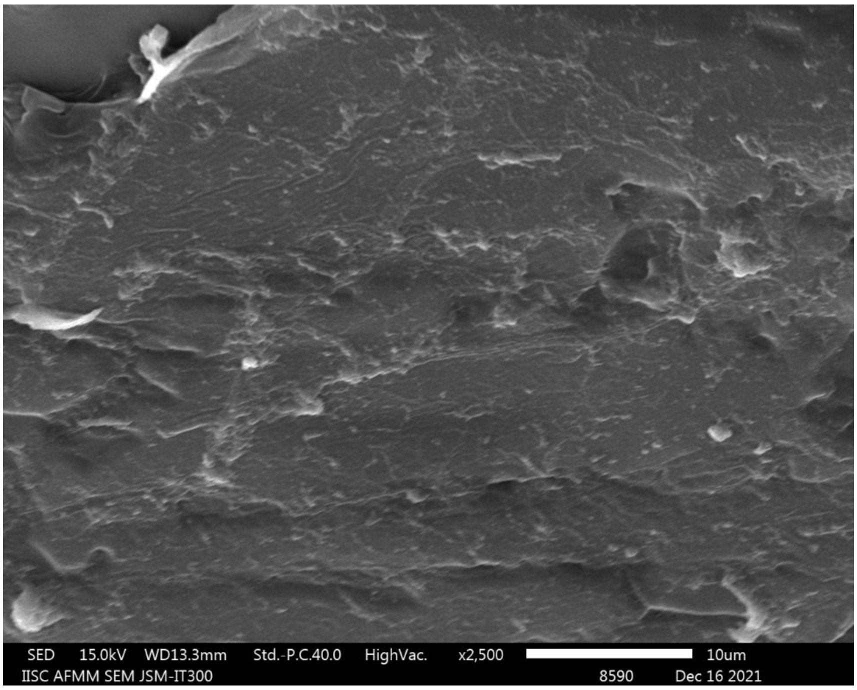

(Da) *C. albicans* S470 Control at 24 h

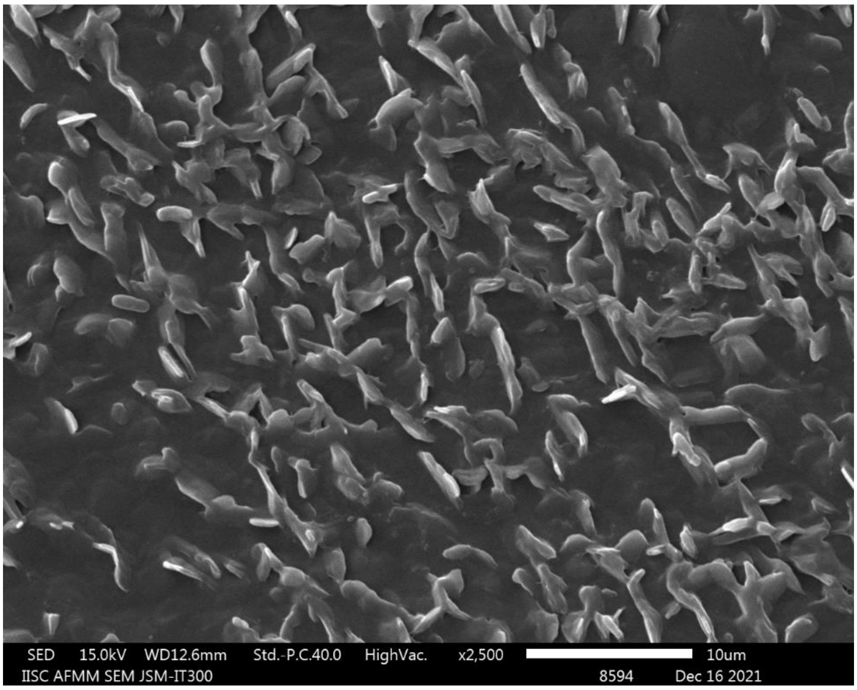

(Db) *C. albicans* S470 Garlic Treated at 24 h

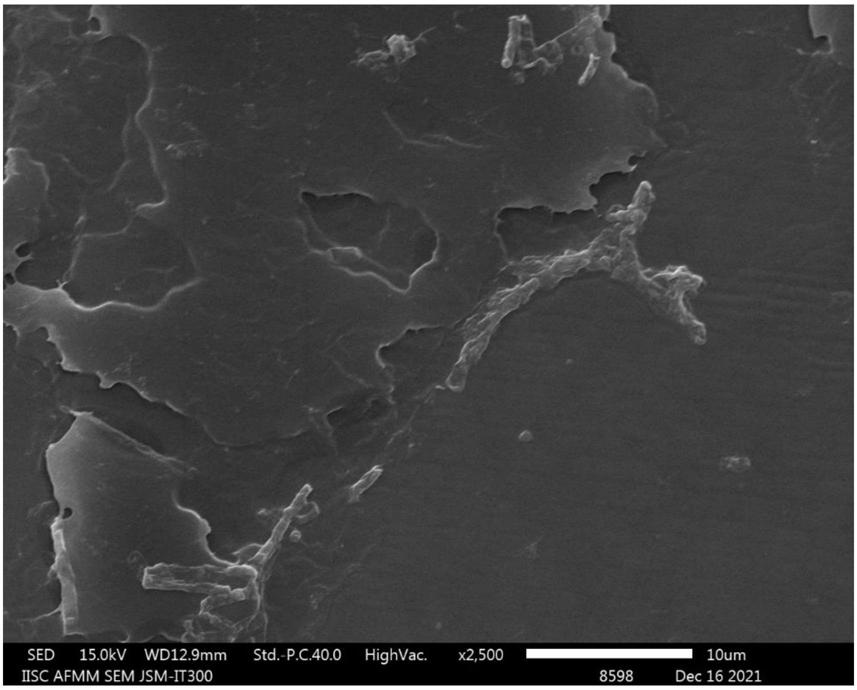

(Dc) *C. albicans* S470 Gooseberry Treated at 24 h

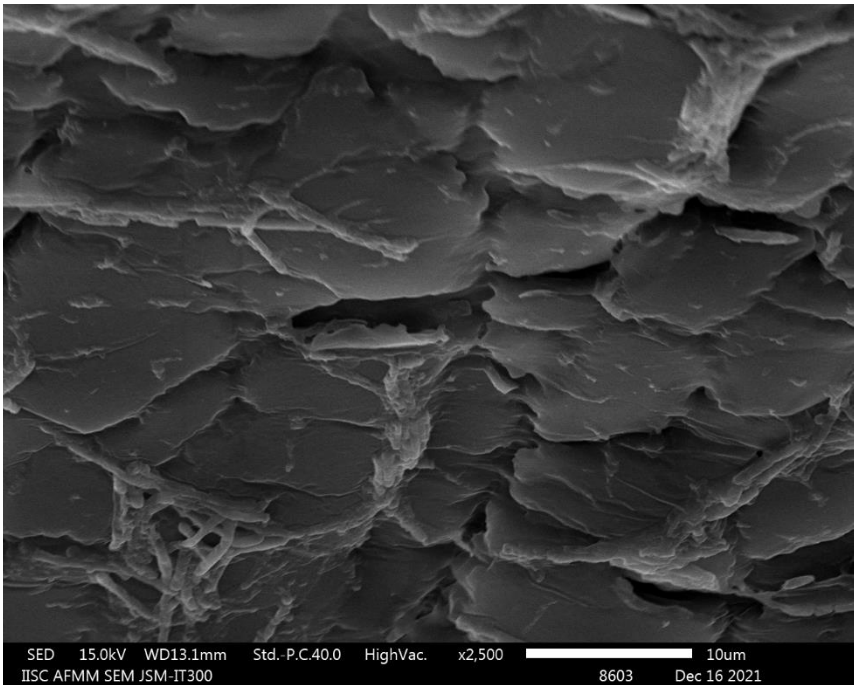

(Dd) *C. albicans* S470 Clove Treated at 24 h

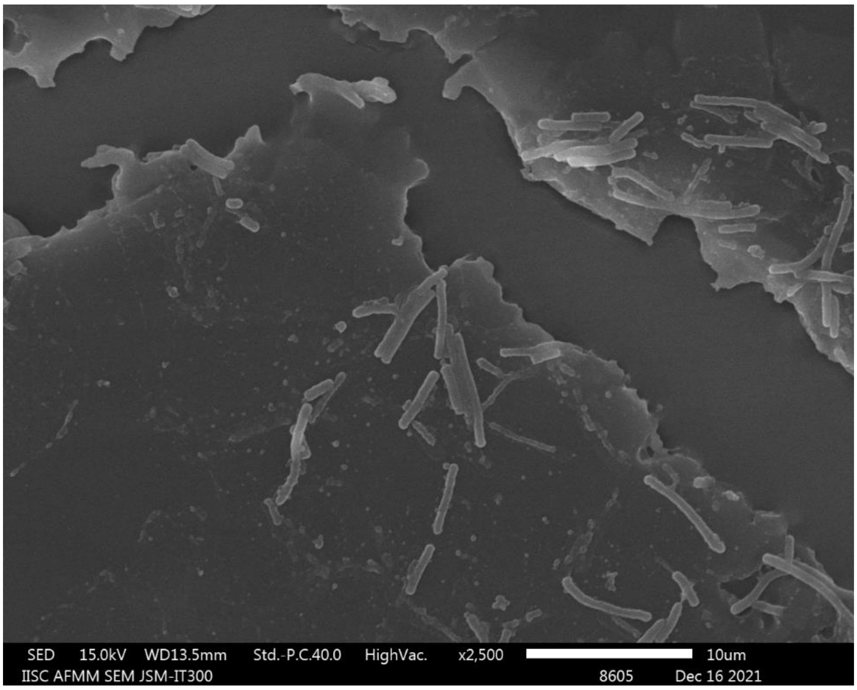

Figure S5

(Aa) *C. albicans* M-207 Control at 12 h

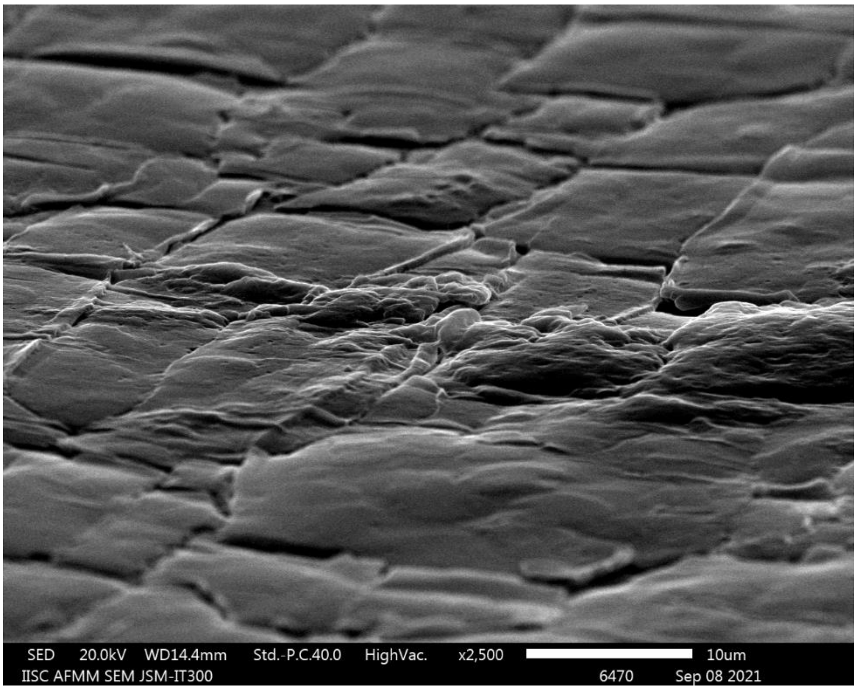

(Ab) *C. albicans* M-207 Garlic Treated at 12 h

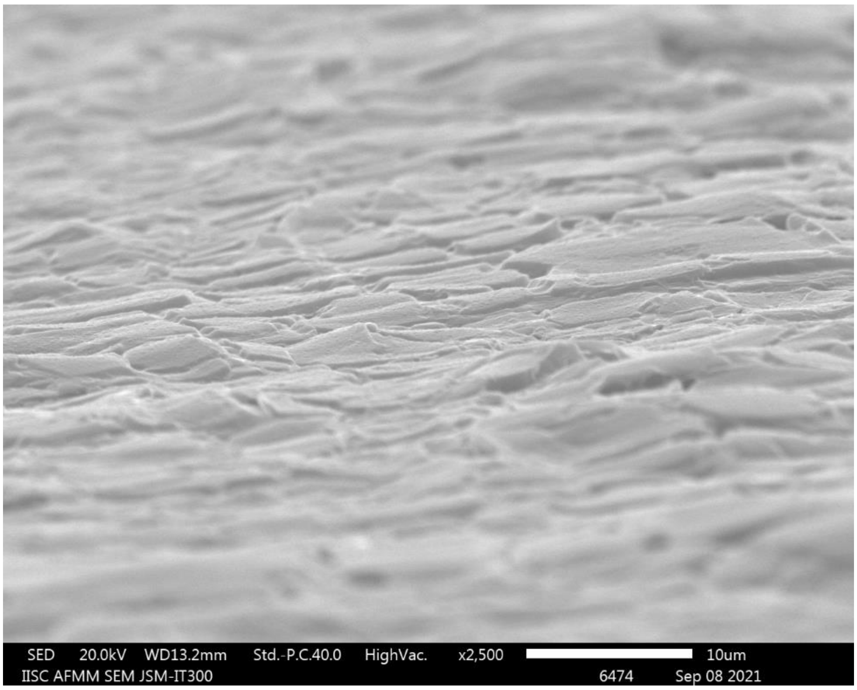

(Ba) *C. albicans* S-470 Control at 12 h

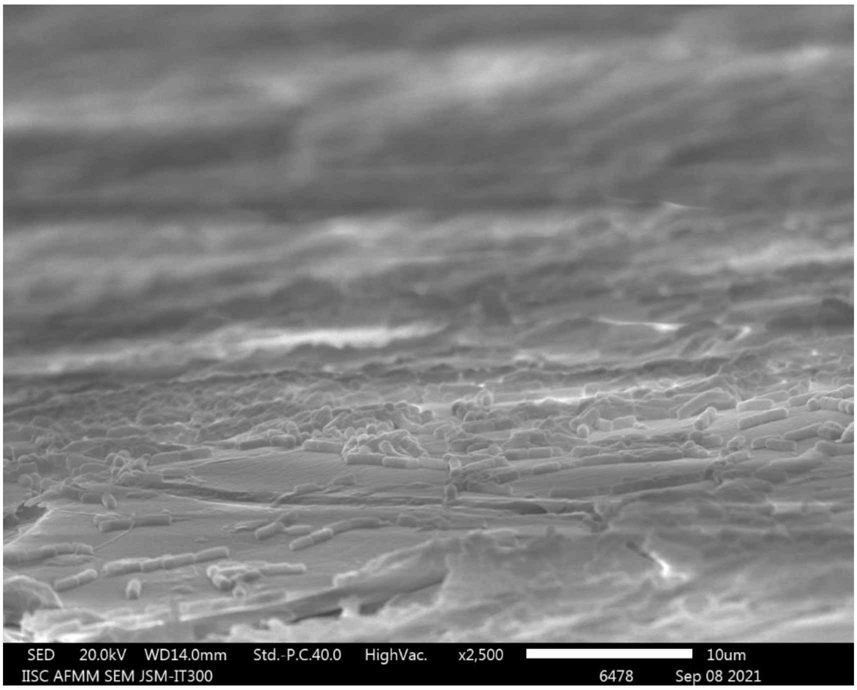

(Bb) *C. albicans* S-470 Garlic Treated at 12 h

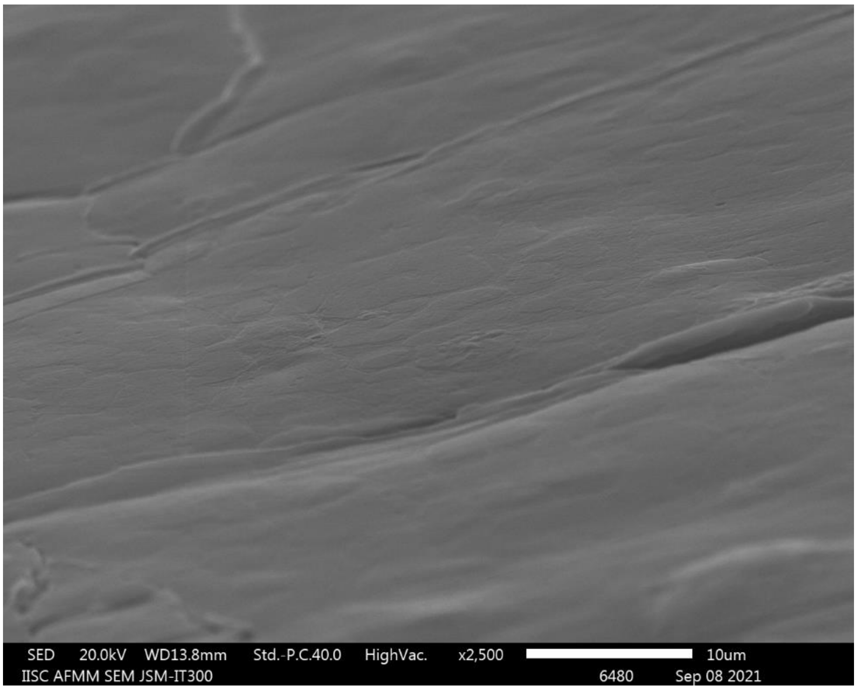

(Bc) *C. albicans* S-470 Gooseberry Treated at 12 h

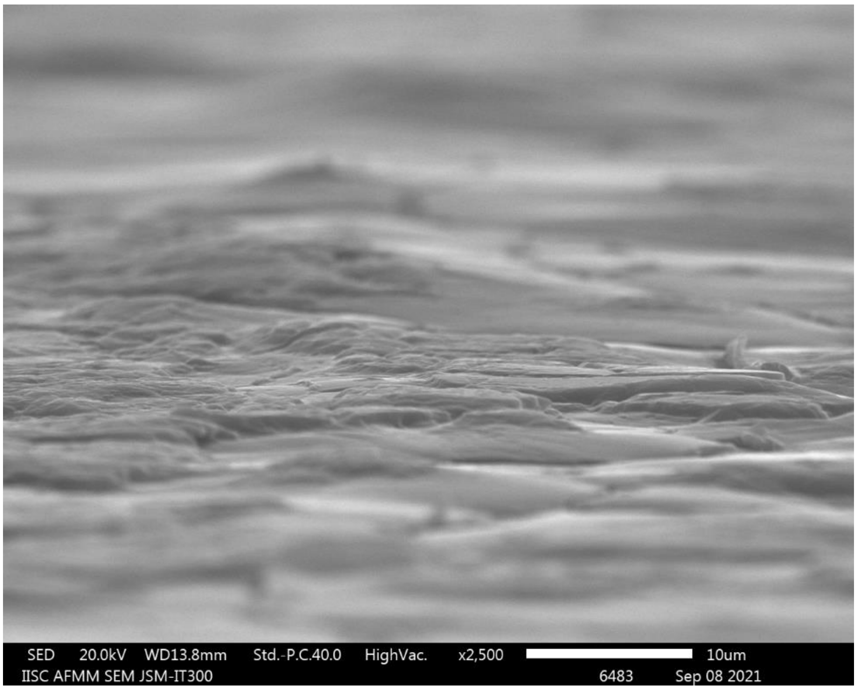

(Bd) *C. albicans* S-470 Clove Treated at 12 h

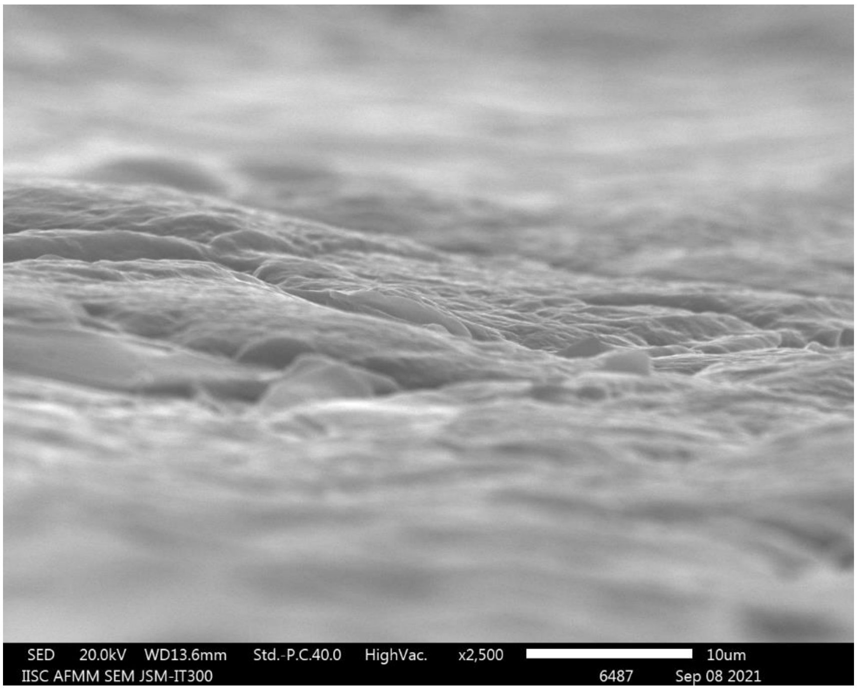

Figure S6

(Aa) Blank

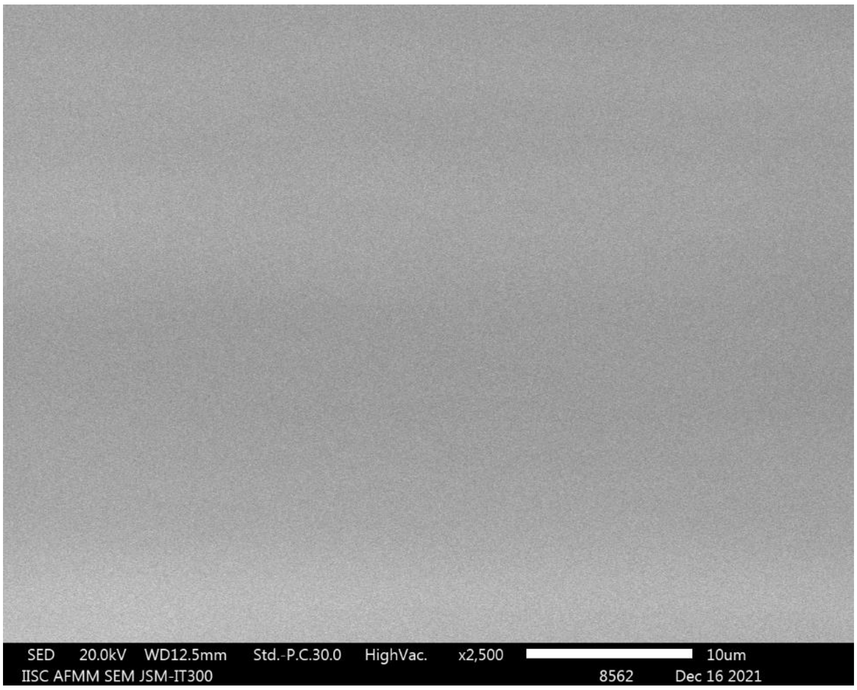

(Ba) *C. albicans* M-207 Control at 24 h

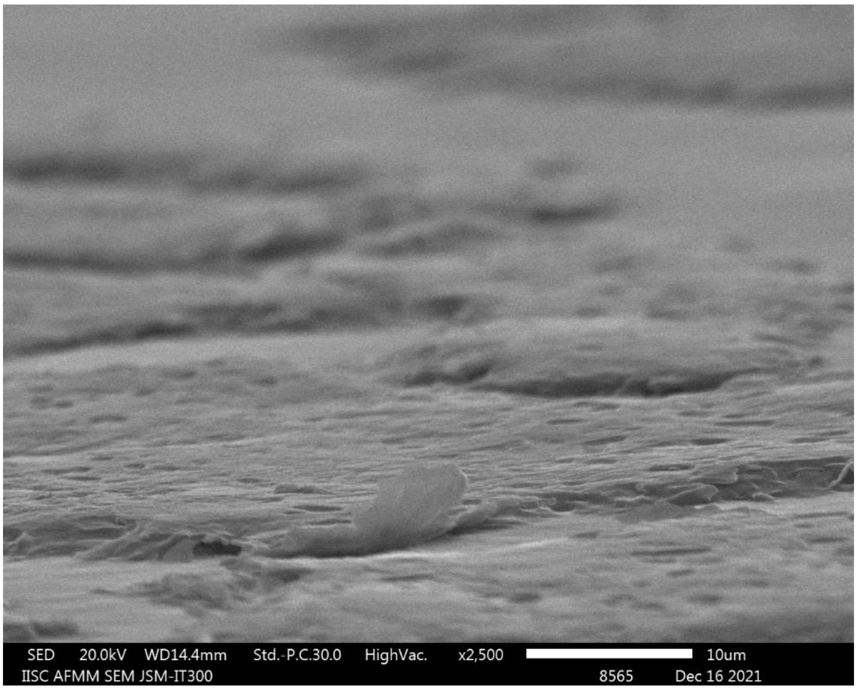

(Bb) *C. albicans* M-207 Garlic Treated at 24 h

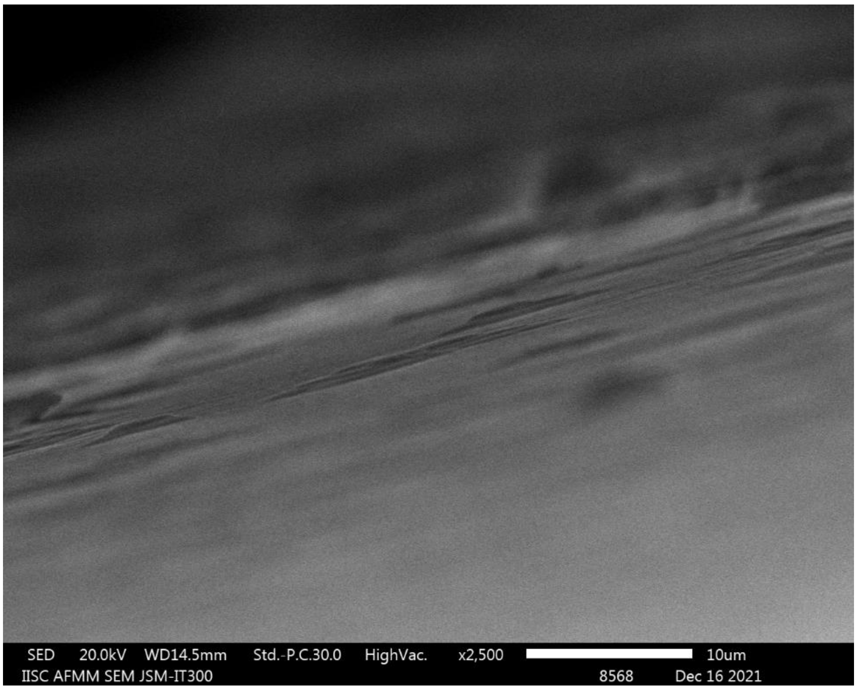

(Ca) *C. albicans* S-470 Control at 24 h

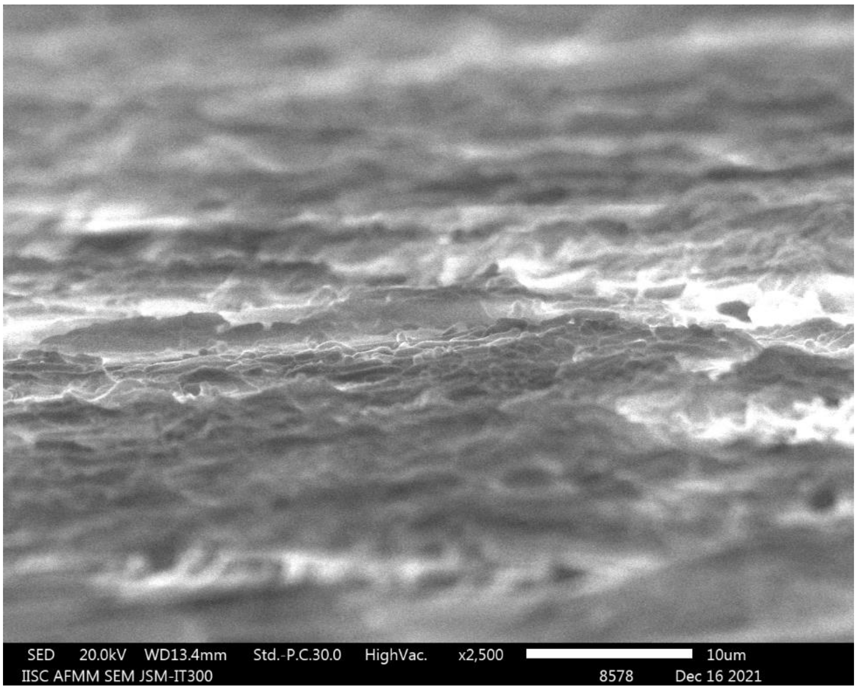

(Cb) *C. albicans* S-470 Garlic Treated at 24 h

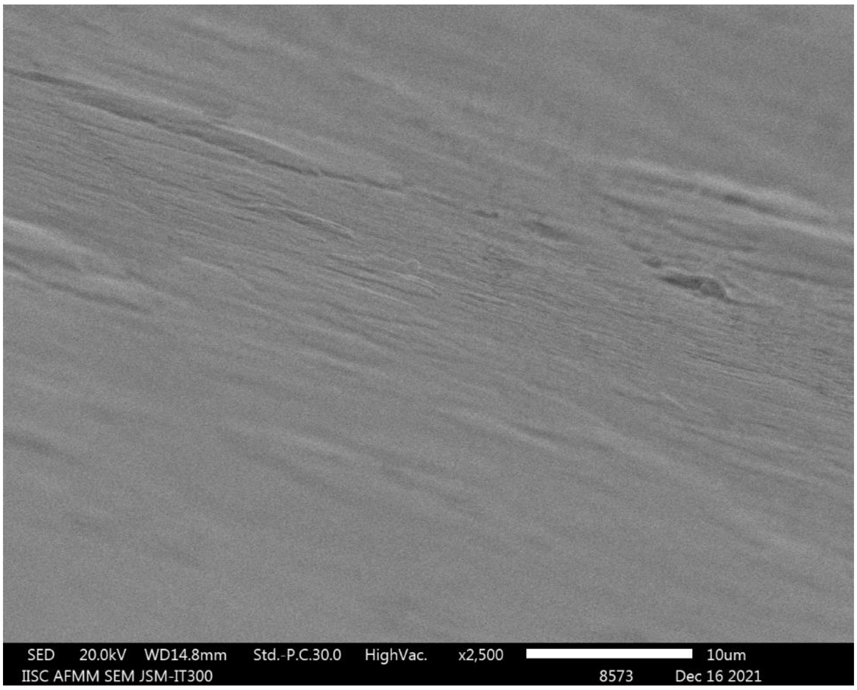

(Cc) *C. albicans* S-470 Gooseberry Treated at 24 h

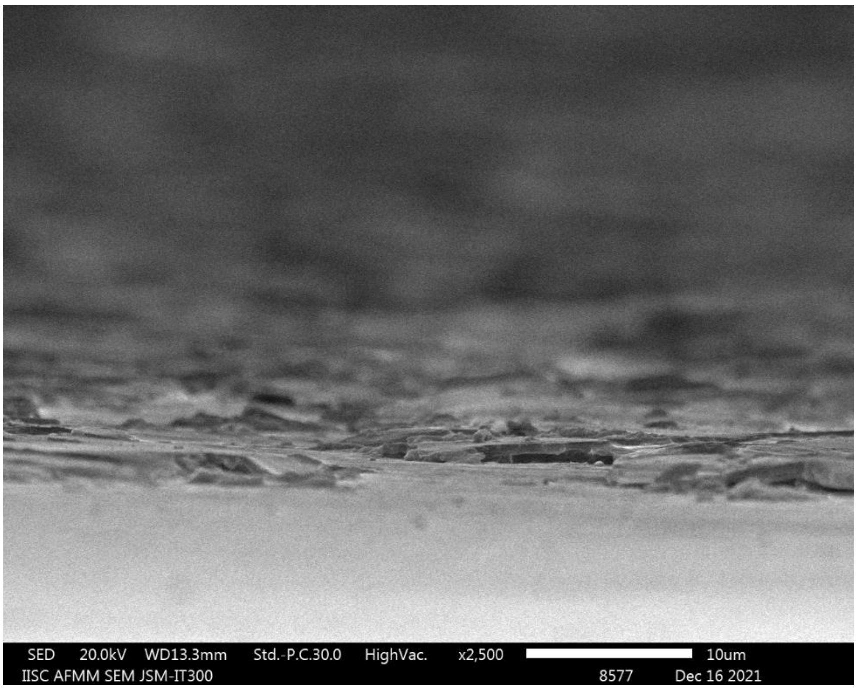

(Cd) *C. albicans* S-470 Clove Treated at 24 h

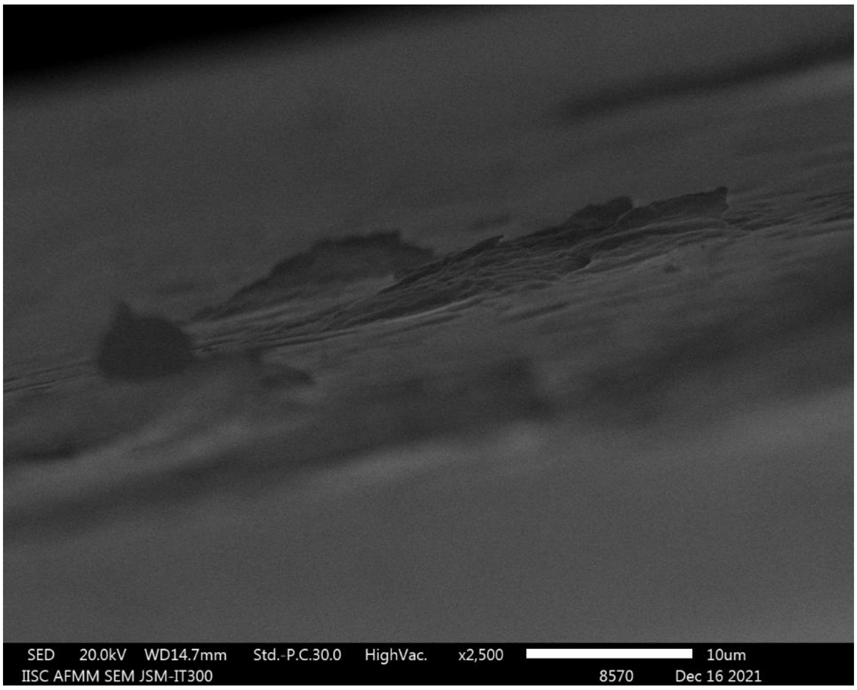

Supplement: Supplementary file 1 [file gels-11-00023-s001.zip › Supplementary material 2.pdf]
